# The Effect of Health on Labour Supply of Rural Elderly People in China—An Empirical Analysis Using CHARLS Data

**DOI:** 10.3390/ijerph16071195

**Published:** 2019-04-03

**Authors:** Jinqi Jiang, Wanzhen Huang, Zhenhua Wang, Guangsheng Zhang

**Affiliations:** 1 College of Economics and Management, Shenyang Agricultural University, Shenyang 110866, China; jiangjinqi812@163.com (J.J.); statasyau@163.com (W.H.); 2 School of Business, Liaoning University, Shenyang 110031, China

**Keywords:** health, labour supply, employment allocation, rural elderly

## Abstract

In China, due to decades of the ‘one-child policy’ and continuous rural-urban labour migration, real population aging in rural areas is increasing more quickly than in urban areas, and the labour inputs in agricultural production are becoming ever more dependent on the elderly. Using CHARLS data, we examine the effect of health on the labour supply of rural elderly people. We construct a latent health stock index (LHSI) to eliminate measurement bias and then use this one-period lagged LHSI and the Heckman two-stage and the Bourguignon-Fournier-Gurand two-stage method to deal with the simultaneous causality of health and labour decisions and sample selectivity in model estimation. The results show that, in the overall level, the labour force participation and work time of rural elderly people increase significantly with the improvement of health. These effects on the males are sharply greater than on the females and are enhanced with age. In the subdivided agricultural and non-agricultural labour supply, health improvement is positively related with labour force participation of rural elderly and brings an employment allocation from agricultural section to non-agricultural section, especially on the males. However, as the work time, these relations are insignificant and invariant with gender and age.

## 1. Introduction

The growing aging population poses a huge challenge to China’s industrialization and modernization. For rural areas, where the population is aging even more than in urban areas, off-farm employment of young workers and their prolonged out-migration to urban areas has further deepened the actual degree of aging and exacerbated its adverse effects on agricultural production and rural economic and social development. Of all of the challenges that the aging population poses for rural China, the aging of the agricultural labour force and the consequent scarcity of high-quality agricultural labour and the shortage of agricultural labour reserve has become one of the most talked about issues in current research. It has triggered a concern throughout Chinese society regarding the question of ‘who will farm in China in the future?’ [1]. The shortage of effective supply of agricultural labour is not only strongly related to the development of agriculture and the rural economy, but also, if this situation persists for a long time, it may weaken the foundation of China’s grain production and food supply, and subsequently, Chinese food security and social stability [2].

However, from the perspective of allocative efficiency, off-farm employment of young rural labour is not only in line with China’s current economic development strategy, but it also supports household income maximization through the division of labour in the family. This means that the labour input in agriculture depends on the long-term effective labour supply and allocation across agricultural and non-agricultural sectors for rural elderly people [3]. Therefore, from this perspective, the realistic needs of China’s social and economic development require us to continue to study the labour supply of rural elderly people and identify the important determinants of their supply decisions through rigorous scientific analysis.

Although age, sex, education, employment opportunities, and wages are important factors of the decision, for the elderly, health is the key determinant [4]. Due to the decline in physical function, the health of elderly people is more vulnerable to the shock of fatigue and illness and their recovery capability after health shocks is weaker. In addition, the depreciation of health accelerates with age [5]. This indicates that health issues are more common and obvious among the elderly. The deterioration of health directly decreases productivity and work capacity, causing them to decide to either shorten their working time and reduce labour intensity or exit the labour market completely. Thus, in theory, the labour supply decision of older people is more susceptible to health variations [6]. And this is even for the rural elderly people in China. However, the relationship is not limited to this. Because of the sharp differences in labour intensity and work environment, there are great differences in the health requirements between agricultural and non-agricultural employment [7]. The effects of health on labour supply for these people are also on the allocation between agricultural and non-agricultural sectors.

Almost all Chinese studies on the effects of health on labour supply appeared after 2000 [8,9,10,11], and most of them explored this effect using rural samples. From the studies using a rural full-sample and out-migration sample, health improvements have a significant positive effect on labour supply [12,13,14,15]. However, for rural elderly people, conclusions are inconsistent. In here, some studies found that there is a ‘ceaseless toil’ phenomenon among elderly people in rural China; that is, they do not significantly reduce labour supply due to health deterioration as they grow older [16,17]. Therefore, it was concluded that health is an insignificant factor on their labour supply. But, some others provided evidences for the significant positive effect of health [1,18,19].

Reviewing the existing literature, we have identified three potential challenges. First, there are limitations to the measures of health used, such as the subjective Self-Assessed Health Status (SAH) or objective medical indicators like Body Mass Index (BMI), Activities of Daily Living (ADLs), Instrumental Activities of Daily Living (IADLs) and chronic diseases, and this may be the reason for the inconsistent findings. For example, in the study of Tan and Zhou, the proxy variable of health, measured by SAH, has no impact on the labour supply of the elderly, while the variable indexed by BMI is strongly significant [17]; Second, the endogeneity that arises from the simultaneous causality of health and labour supply decisions and the sample selection in labour supply is not fully considered in the estimation, and this could also be a source of biased regression results. Third, age and gender variation in the relationship between health and the labour supply decision and the difference of these relations between agricultural and non-agricultural employment are not discussed in-depth within an identical framework.

In light of the above three challenges, this paper attempts to complete two innovations. On the methodology, after analysing the advantages and disadvantages of the existing measurements of health and endogeneity in the econometric estimation of the health and labour supply, we construct a latent health stock index (LHSI), as presented by Bound et al. [20] and Disney et al. [21], and then comprehensively use the one-period lagged health index, the Heckman two-stage method (Heckman method), and the Bourguignon-Fournier-Gurand two-stage method (BFG method) in the estimation to ensure the effectiveness and reliability of the results. On the content, we examine the overall impact of health on the rural elderly labour supply. Furthermore, we explore the role of health in agricultural and non-agricultural sectors and the gender and age differences of health effects.

The remainder of this paper is structured as follows: the regression model, the definition of variables, and the data description are explained in Section 2. Then, we present the empirical results in Section 3, and we conclude with the findings and consider the implications of our work in Section 4.

## 2. Model, Estimation Method, and Data

### 2.1. Models and Estimation Method

#### 2.1.1. Regression Models

The labour supply decision is generally considered to be a two-stage decision-making process. In the first stage, the individual decides whether to participate in the labour market and what type of job to work, that is, the labour force participation (LFP) decision. In the second stage, after deciding to work, the individual chooses how much labour time to provide, that is, the labour time decision. And in this paper, LFP is defined as the occurrence of some working behaviours in a certain period of time; labour time is defined as the individuals’ total working time in the labour market during a certain period of time. In the empirical studies, scholars set up the corresponding regression model in line with the decision characteristics of these two stages and then identify the important factors affecting each stage of the supply decisions. In this paper, we also use the two-stage decision model to explore the impact of health on labour supply of rural elderly people. And the regression equations used for the first stage decision are:

(1) $$prob(y=1|H,Χ)=F(\alpha +\beta H+\sum \gamma_{j}{Χ}_{j})$$

(2) $$prob(y=i|H,X)=F\left(\alpha +\beta H+\sum \gamma_{j}{Χ}_{j}\right),i=0,1,2,3$$

where, Equation (1) is for the decision of whether to work, and Equation (2) is for the decision of what type job to accept. They are discrete choices regression models, but Equation (1) is a binary choice model where the value of the dependent variable is 0 when the individual does not work, and it is 1 when the individual works. Equation (2) is a categorical choice model where a value of 0 means the individual does not work; 1 means the individual works in agriculture; 2 means the individual has off-farm fulltime employment, and 3 means the individual is involved in fulltime off-farm self- employment. H is a proxy variable for health status, and $X_{j}$ represents the control variables (i.e., age, sex, education, etc). A detailed description of the variables can be found in the Section 2.2.2. According to the data type of the dependent variable in Equations (1) and (2), we use logit and multinomial logit regressions, respectively.

A labour time decision model is used for the empirical analysis of the second stage decision, and regression equation is:

(3) $$time=\alpha +\beta H+\sum \gamma_{j}X_{j}$$

This model is estimated on the sample of individuals who are employed. The dependent variable time in Equation (3) is working time and it refers to labour time regardless of production, agricultural labour time, non-agricultural full-time employment labour time, and non-agricultural full-time self-employment labour time, respectively, in the regression. The independent variables in Equation (3) are the same as those in Equations (1) and (2). Since the working time is a continuous variable, we use the Ordinary Least Squares method for estimation.

#### 2.1.2. Endogeneity and Econometric Approaches

In this part, we have a detailed discussion of the endogenous problems in the existing studies regarding the health effects on the labour supply and explain their corresponding estimation strategies. The first is the measurement error of health. There are two categories of measures of health used in this field: subjective measures, such as SAH, and objective measures such as ADLs, IADLs, BMI and so on. Due to its level of comprehensiveness and the presence of private information unable to be effectively captured through objective measures [10,22], SAH is most widely used measure of health. However, SAH often has measurement errors [21] because of ‘justification bias’ (since poor health may represent a legitimate reason to be economically inactive, individuals who are not working may report worse-than-actual health as a way to rationalize behaviour. Also, individuals with lower financial rewards of continuing in the labour market have an incentive to report poor health as means of obtaining disability benefits.) [10,23], the subjective judgments of health which may not be comparable across individuals (objective medical measures generally just infer one or a few dimensions of health, and thus health variables are less comprehensive than SAH) [24]. Objective health variables such as ADLs, IADLs, and BMI are medical indicators that can be accurately measured or observed externally. Although they can effectively avoid the measurement error of SAH, they commonly have ‘information loss’ (for example, ADLs are significantly different among older populations, but there is very small difference in younger populations. BMI is determined by weight and height, and it difficult to change for adult individuals. Therefore, it is generally suitable for health analysis in children and adolescents) [12,22,25], they demonstrate limited applicability for all age groups, and pose a certain level of difficulty in explaining regression results.

Comparing the advantages and disadvantages of the above two types of health measures, we still use SAH as a proxy variable of health. To eliminate SAH measurement errors as much as possible, we adapt the strategy used in Bound et al. [20] and Disney et al. [21] to construct a latent stock index of health for each individual as a function of personal characteristics and objective health indicators. This constructed variable is used to indicate the real health status in the following estimation. The latent health stock constructing model (LSAH) is formed as:

(4) $$SAH=\Phi \left(OH_{1},OH_{2},\ldots ,Y\right)$$

where $OH_{i}$ are detailed objective health measures (i.e., ADLs, chronic disease, functional disabilities, mental depression, physical pain or discomfort, and other diseases in this paper). A detailed description of these objective health variablesis listed in Table A1. Y are the personal characteristics of the individual (i.e., age, sex, education level, and the region of residence).

The second challenge is the selectivity of labour supply of rural elders. For simple binary self-selection problems, scholars usually use the Heckman method (the Heckman two-step model) for estimation. This study also adopts the Heckman method, but we know that self-selection in labour supply is not only the choice of whether to work but also the choice of what jobs to work, and these lead to the multivariate endogenous regressors as well as the binary endogenous regressors in the estimation of Equation (3). For this regression with multivariate endogenous regressors, we use the BFG method, first introduced in Bourguignon et al. [26].

The final issue is the simultaneous correlations between health status and labour supply. The simultaneous causality of health and labour supply was first elaborated in Grossman [5] and further confirmed in the theoretical study of Pitt and Rosenzweig [27] this number used above for a different reference. Strong evidence from subsequent empirical research further supported these works [21,28,29]. For the simultaneous correlations problem, the simultaneous equations model (SEMs), regression discontinuity method (RD), and model with a lagged health variable were the major estimation approaches in early studies. In this study, we introduce the one-period lagged LHSI in Equations (1)–(3) as a proxy variable for health.

### 2.2. Data Sources and Variable Definitions

#### 2.2.1. Data Sources

The data used in this paper are from the China Health and Retirement Longitudinal Study (CHARLS). The CHARLS is a set of high-quality micro-survey data comprised of Chinese adults aged 45 and over and their family members. The first wave of this survey was conducted in 2011, and it has been repeated every two years. In this paper, we use the rural sample.

Before analysing the data, we first exclude the individuals under the age of 45 and those for whom age is missing. Then, we remove the individuals who have missing values for the variables of employment, SAH, objective health indicators, wage, household income, pension, and so on. Finally, we drop any outliers with working time of more than 6000 h per year. After data cleaning, the entire sample is composed of 40,271 rural elderly individuals: 13,427 observers in 2011, 13,995 observers in 2013, and 12,849 observers in 2015. The samples of 2011 and 2013 are used to obtain the lagged LSHI.

#### 2.2.2. Variable Definition and Description

According to the employment definition of the National Bureau of Statistics of China, if an individual’s weekly working time is less than one hour on average, he/she is considered not in work. When his/her working hours average more than 1 h per week and the total working time is more than 52 h in a year, he/she has labour supply [10]. With this definition, we determine the individuals’ working behaviours are considered as statistical labour supply using their answers on questions such as: ‘*Did you have farming employment, self-agricultural production, off-farm employment or off-farm self-employment more than 10 days in last year*?’; ‘*How many months did you work in a year?*’; ‘*How many days did you work in a week?*’; and ‘*How many hours did you work in a day?*’. Furthermore, we identify whether they belong to the market labour supply with the answers to the questions of ‘*Did you earn wage?*’ or ‘*Did you have economic revenue?*’, and in this case, housework is not considered to be labour supply. As previously noted, we consider labour supply to be a two-step decision process based on the occurrence of labour force participation and then labour time. If an individual has labour supply in the market, we defined the dependent variable value in Equation (1) as 1, otherwise as 0. Labour supply is further subdivided into unsupplied, agricultural labour supply, non-agricultural employment supply, and non-agricultural self-employment supply in Equation (2). The labour time is the total yearly working hours, equal to the working months per a year × working days per a week × working hours per a day × (30/7).

For health, we used the LHSI constructed from Equation (4). The control variables in Equations (1)–(4) are classified in five categories: (1) individual demographic characteristics like gender, age (and its square), education level, and marital status; (2) social security status (We have included the new cooperative medical system (NCMS) as the variable of social medical insurance status in the pre-regression. Since the NCMS achieved full coverage of rural people in 2011, the NCMS variable has very little variation, and the multinomial logit regression cannot obtain convergence. Therefore, we exclude this variable in all regressions.), mainly the pension status; (3) household production and economic status, containing the variables of the presence of farmland or water aquaculture, the total area of farmland and water aquaculture, ownership of agricultural machinery, household per capita consumption, and the proportion of off-farm income; (4) regional dummy variables; and (5) objective health indicators, including ADLs, chronic diseases, functional disabilities, mental depression, physical pain or discomfort, and other diseases. Table 1 is the definition and value description of the variables.

## 3. The Empirical Results

### 3.1. Health and LFP of the Rural Elderly People

#### 3.1.1. Health and the Overall LFP in all Employments

Beginning with the original health variable SAH, we use a logit regression for Equation (1) to estimate the effect of health on the labour force participation of rural elderly people and consider this as a benchmark (see Column 1 in Table 2). In this benchmark regression, we handle this ordinal variable of health as a continuous variable. Excluding the benchmark estimation in Table 2, Column 1, we use the one-period lagged LHSI as the explanatory variable in all regressions in Table 2, Table 3, Table 4 and Table 5. Comparing the baseline coefficient of SAH in Column 1, we find that the absolute value of the coefficient of the one-period lagged LHSI in Column 2 has increased significantly. This confirms the conclusion that the measurement error would underestimate the impact of health on labour supply and supports using the LHSI approach to increase the effectiveness and reliability of the estimation results [21]. We explain the outcomes by the regressions with LHSI in the following sections.

In Table 2, Column 2, because, in the CHARLS data, a higher value of SAH means poorer health, while a lower value means better health, the LHSI has a negative coefficient and passes the Z test at the 1% significance level, indicating that health is indeed an important explanatory factor of the labour supply of rural elderly people and confirming a positive relationship between good health and labour supply. This means that these rural elderly people with good health stock have a greater likelihood of working, while those with poor health are less likely to work. To further explore the magnitude of the health impacts, we calculated the marginal effect of health on the possibility of labour supply. The marginal effects here and below are the marginal effect at a mean level of health. The results show that, given the conditions of the personal demographic, household production, and economic characteristics, the labour supply probability of rural older people will increase by 8.72 percentage points, on average, with improvements in health. This effect is distinctly higher than the value of 3.48 percentage points found in Liu [12]. From this marginal effect, health has a notable impact on rural elderly people’s labour supply decision. With the continued improvement of medical and health conditions, better living standards, and the widening and deepening of medical insurance coverage, the health status of rural elderly people will continue to improve. For rural areas, where aging is rapidly deepening and the shortage of the high-quality labour inputs in agriculture is becoming more severe, if we can optimize the labour supply potential of rural older people by guaranteeing health conditions, it will be very beneficial.

#### 3.1.2. Health and the Type of LFP

Classifying labour force participation decisions as non-participation, agricultural labour supply, off-farm employment, and off-farm self-employment, we further investigate the effect of health on the labour force participation of rural elders. The outcomes in Columns 3–5 of Table 2 are the results of the multinomial logit regression of Equation (2). From these results, we find that health has a distinct impact on all three types of labour supply for rural older people and people with good health have high probabilities of LFP in agricultural and non-agricultural production. Specifically, the marginal effect of health on rural older people’s possibilities for agricultural employment, non-agricultural employment, and non-agricultural self-employment are 2.10 per cent, 15.59 per cent, and 10.74 per cent, respectively. Given the marginal effect, health improvements for the rural elderly could result in higher probabilities of participation in off-farm employment and off-farm self-employment, although it has a less increasing effect on agricultural employment. Based on these results, we should be aware of the fact that increased work capacity of rural elderly people due to health improvements is more likely to be allocated to the second and third sectors, while agriculture is still at a disadvantaged position in terms of labour allocation.

### 3.2. Health and Working Time of the Rural Elderly People

#### 3.2.1. Health and Overall Working Time

After the analysis of the health effect on LFP, we further empirically explore the impact of health on the labour supply time of the elderly in rural areas. Here, we first use the Heckman method to estimate the health effect on individuals’ overall labour time and conduct a statistical test on the Inverse Mills Ratio (IMR) to determine the existence of self-selectivity within the sample. From the IMR results, it passed the test at a 5% significance level, confirming the existence of the sample self-selection problem. Therefore, we provide the results of the overall working time using the Heckman method (Column 1 in Table 3) and the sub-divided working time on the types of production using the BFG method (Column 2–4 in Table 3). Different from the regression of LFP in Table 2, we substitute the variable of household ownership of arable land or aquaculture water surface with the total area of arable land or aquaculture water surface and exclude the variable of the proportion of off-farm income in the estimation of Equation (3).

From the results of overall working time in Column 1 of Table 3, the estimated coefficient of health is negative and significant at the 1% level. This shows that, regardless of the employment type, good health status has a significant positive effect not only on labour participation, but also on labour time. The coefficient of −149.2 indicates that, given the other conditions are constant, the marginal yearly working time would increase 149.2 h with an improvement in health. Calculated using a standard working time of 8 h per day, the total labour time of rural older people would decline by 18.65 days per year.

In addition, in terms of working time as a whole, important factors also include gender, pension status, and the regional dummy variables. Elderly females have significantly shorter working hours than their male counterparts, and those who have pensions are more likely to participate in the labour market, but their labour hours are less. The working hours of the elderly in north-eastern and central China are significantly lower than those in the western region, while the eastern region is indifferent to those in western region.

#### 3.2.2. Health and Working Time among Different Types of Employment

Considering the labour supply of the rural elderly can be divided into agricultural employment, non-agricultural employment, and non-agricultural self-employment, we list the results of these three types of subdivided working time in Column 2–4 of Table 3.

From the results in Column 1 and Columns 2–4 of Table 3, the estimation results in the latter are very different from the former. For health, it does not pass the significance test in all three types of working time, and it indicates that good health status does not lead to an obviously longer working time in each employment population. This result is opposite to the findings of Wang and Liu (2016). Based on these results, and combined with the health effect on the overall LFP and overall labour time, we think the health impact on the labour supply of rural elderly people is mainly reflective of the decision to work or no. With respect to labour time, its effect was reflected in the average working time between the three different types of employment, and there is no significant explanation for the difference in labour time within each type.

### 3.3. The Gender and Age Differences of the Health Effect

#### 3.3.1. Differences in LFP

Regression results in the above section demonstrate that the labour supply of the rural elderly has obvious gender differences and also varies sharply with increasing age. Therefore, we estimate the impact of health among gender and age groups in this section. Table 4 illustrates the gender and age differences in LFP. Due to space limitations, we have only listed the estimated coefficients and marginal effects of health in Table 4 and Table 5.

From the results of gender grouping, the health variable passed the test at the 1% significance level in all regressions. The negative values indicate that health has a significant positive effect on the overall LFP and the sub-divided participation in agricultural employment, non-agricultural employment, and non-agricultural self-employment of both rural males and females. Since the variable’s coefficients in logit regression are incomparable [30], we use the marginal effects (i.e., the ME in Table 4) to state the gender differences in health effects. In terms of overall participation, the probability of LFP among rural males and females would increase 8.74 per cent and 8.69 per cent, respectively, due to health improvements. These differences among gender groups are very inconspicuous.

**Figure 1:**
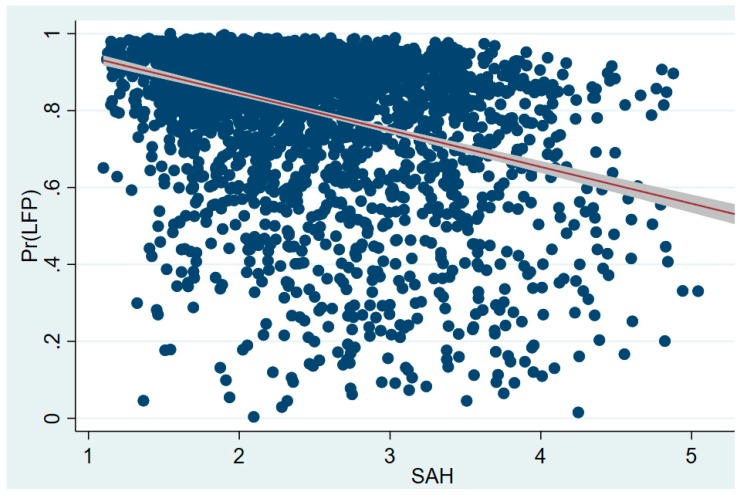
Health and LFP of rural elderly men.

However, upon review of the sub-divided labour supply, we find that there are some gender differences. Firstly, in agricultural employment, even the positive marginal effect in male group means males in good health have less probability of labour participation at the mean value of health. We still believe that, from the scatter plot of the health and the probability of participation (Figure 1) and the coefficient of health, health improvements would increase the probability of LFP in agriculture of rural elderly men. In the female group, the coefficient and the marginal effect of health on its mean are negative, which means a positive impact of health on LFP in agriculture for rural elderly women. As the marginal effect values of health in the male and female groups are small, we believe that, even though health is a significant explanatory variable of male and female elders’ agricultural LFP, the actual effect is relatively weak. Secondly, in non-agricultural employment, marginal health improvements are able to play a substantial role in increasing the possibility of rural male and female elders’ LFP, and this effect in the male group is larger than in the female group. Finally, in non-agricultural self-employment, health improvements also play a role in increasing rural male and female elders’ LFP, but these health effects are relatively weak, and the marginal effect in the male group is smaller than it is in the female group.

From the age grouping results, the health variable passed the test at the 1% significance level in all regressions, and the negative coefficients show that, in all age groups, both in the overall LFP and sub-divided LFP, health improvements have increasing impacts. Among the different age groups, with increasing age, the marginal impact of health improvements on the overall LFP has an upward trend, and its value rises from 5.21 per cent in the 45–50 years old group to 11.09 per cent in the group over 70, reflecting a doubling of the marginal effects. For agricultural employment, we found that good health leads to a higher possibility of LFP, but on the marginal impact among different age groups, it is negative when individuals are 65 years old or less and becomes positive when they older than 65 years old. The marginal effect in the over-70 age group is doubled compared to the 66–70 years old age group.

For non-agricultural employment and non-agricultural self-employment, due to the fact that off-farm job opportunities for rural labour rapidly decline with age and employment tends to shift from non-agricultural sectors to agriculture when they are older, even the negative coefficients mean good health corresponds to a higher probability of LFP in these types of employment. The variations of the marginal effect indicate that the LFP probability of off-farm employment increasing from health improvements rises with age before individuals are 60 years old and then declines, and the LFP probability of off-farm self-employment increases before 55 years old and then decreases.

#### 3.3.2. Differences in Working Time

Based on the rural working elderly, we investigate the age and gender differences of the effect of health on working time and list the results in Table 5. Looking at the gender comparison in overall working time, the rural male and female elderly in good health all have longer labour times than those with bad health. The coefficients of health show that the labour times of the rural males would increase by 163.4 h per year (about 20.43 days) and rural females would increase by 106.9 h per year (about 13.36 days) for every unit of improvement in health. Moreover, health has a significantly greater impact on labour time for males than for females. In the sub-division of employment, we find that, except in the agricultural employment of rural female elders, health is an insignificant factor of working times for rural older men and women in agricultural employment, non-agricultural employment, and non-agricultural self-employment. Based on the above, we consider that the gender differences in the effect of health on working time are mainly reflected in the gender differences of inter-employment type.

Then, looking at the comparison of age groups in the context of overall employment, health has an important impact on working time among rural individuals younger than 66 years old, and its impact increases with age. However, for those individuals older than 66 years old, health is insignificant in the estimation. In the sub-divided employment categories, the regression results show that the impact of health on labour time becomes insignificant in all age groups. Therefore, we consider that the age differences in the effect of health on working time are mainly reflected in the age differences of inter-employment type.

## 4. Conclusions

We use CHARLS data to examine the effect of health on the labour supply decision of the rural elderly in China and its gender and age differences. Given the measurement bias of the global health index, the simultaneous causality of health and labour supply decisions, and the sample selection in the labour supply, we construct an LHSI to eliminate measurement bias and then comprehensively use the one-period lagged LHSI and the Heckman and BFG methods to deal with the simultaneous causality and sample selectivity in model estimation. Our results show that:

(1) Health has a significantly positive impact on the overall probability of LFP. With other conditions constant, the LFP probability would increase by 8.72 per cent, on average, marginally with improvements in health.
(2) In the sub-divided employment types, health has a significantly positive influence on the probability of LFP in agricultural employment, off-farm employment, and off-farm self-employment. The marginal effect from improvement in health across these three types employments are 2.10 per cent, 15.59 per cent and 10.74 per cent, respectively, reflecting a stronger impact on LFP in off-farm employment than it in agricultural employment.
(3) With regards to working time, health improvements have a significant increasing effect. Holding other factors constant, the marginal effect of health is, on average, 149.2 h per year (about 18.65 days).
(4) In the sub-divided employment types, health is insignificant for working time in all three types of employment.
(5) From the gender comparison on LFP, we believe that the health condition changes can significantly affect the LFP of rural male and female elders. As far as the degree of impact is concerned, health effects on the LFP for non-agricultural employment are significantly greater in males than in females, while the impacts on males in agricultural employment and non-agricultural self-employment are slightly less than those on females.
(6) From the gender comparison on labour time, health has a strong positive effect on overall working time, and the influence on males is significantly larger than females. However, in the sub-divided employment types, this effect of health becomes insignificant in both male and female groups.
(7) From the age comparison on LFP, improvements in health have a positive impact on overall LFP which increases with age. However, for agricultural employment, even though good health leads to a higher possibility of LFP, the marginal impact is negative when individuals are 65 years old or younger and positive when they are older than 65 years old. For off-farm employment and off-farm self-employment, good health corresponds with a higher probability of LFP in these two types of employment and the marginal effect from health improvements all experience an inverse U-type process that rises at first and then decreases with increases in age.
(8) From the age comparison on labour time, health has a significant impact on overall working time when rural individuals are less than 66 years old and its impact increases with age. However, in the sub-divided employment types, this impact becomes insignificant in all age groups.

Based on these findings, we believe that the assertion of the ‘ceaseless toil’ for rural elders in China is hardly sustained empirically. For older people in rural areas, although the phenomenon that they are still working after the age of 60 is more common than among their urban counterparts, the results show that the increasing marginal effect of health on the labour supply with age growing have oppose the concept of the ‘ceaseless toil’. Meanwhile, given that agricultural labour input is increasingly dependent on the supply of rural elderly labour due to the integrated effect of population aging and continuous out-migration from agriculture for rural young labour, effective measures to improve the health of rural elderly people are key to alleviating the labour input shortage and ensuring production stability China’s agricultural sector. However, in policy design, the conflict between labour supply and health welfare of elders requires the governments to coordinate their relationship and clarify the key population by policies guiding. Our research proposes a focus on rural elders under the age of 66 as the focus of policy guidance, and their labour supply capacity guaranteed by health needs to be scientifically evaluated. And this would be future work in our researches.

## Figures and Tables

**Table 1:** Variable definition and value description.

Variable Name	Definitions		Value Description		Mean	S.D.
Labour supply						
employ	has LFP		0 no, 1 yes		0.712	0.453
employsup	what type of LFP		0 not work, 1 agricultural production, 2 non-agricultural employment, 3 non-agricultural self-employment		1.060	0.888
worktime	annual total working hours				1459.536	1183.822
agrtime	annual working hours in agriculture				1209.436	1057.804
nagretime	annual working hours in off-farm employment				1848.092	1151.901
snagrtime	annual working hours in off-farm self-employment				1983.734	1471.852
Health						
sah	self-assessed health	1 very good, 2 good, 3 fair, 4 poor, 5 very poor		2.967	0.991	
psah	one-period lagged LHSI			2.981	0.744	
Other variables						
age	age			61.303	9.627	
agesq	square of age			3850.721	124.024	
gender	gender	1 male, 2 female		1.531	0.499	
edu	educational level	1 illiterate, 2 literate, 3 elementary school, 4 junior high school, 5 high school or secondary school, 6 college and above		1.813	1.191	
marry	has spouse	0 no or never married, 1 yes		0.855	0.352	
ifpension	has pension	0 no, 1 yes		0.781	0.414	
ifland	has farmland/aquaculture water	0 no, 1 yes		0.397	0.489	
landarea	total area of farmland and aquaculture water			4.676	49.216	
ifmachine	has agricultural machinery	0 no, 1 yes		0.620	0.485	
pexp	household consumption per capita			4559.975	13,440.03	
nagrincr	the proportion of off-farm income			0.878	1.095	
region	regional dummy variable	1 Western, 2 North-eastern, 3 Central, 4 Eastern		2.597	1.184	

**Table 2:** The regression results of LFP of rural elderly people.

Variables	Logit Regression		Multinomial Logit Regression		
	(1) Benchmark Regression	(2) Overall Supply	(3) Agricultural Employment	(4) Non-Agricultural Employment	(5) Non-Agricultural Self-Employment
sah/psah	−0.345 ***	−0.604 ***	−0.494 ***	−0.901 ***	−0.793 ***
	(0.0281)	(0.0357)	(0.0373)	(0.0515)	(0.0661)
age	1.218 ***	1.457 ***	1.999 ***	3.381 ***	−0.453
	(0.355)	(0.372)	(0.390)	(0.626)	(0.685)
agesq	−0.159 ***	−0.175 ***	−0.199 ***	−0.382 ***	−0.0517
	(0.0279)	(0.0292)	(0.0306)	(0.0518)	(0.0561)
gender= 2	−0.860 ***	−0.795 ***	−0.428 ***	−1.599 ***	−1.248 ***
	(0.0568)	(0.0589)	(0.0626)	(0.0758)	(0.0947)
edu= 2	0.0702	0.0820	0.0960	0.0781	0.112
	(0.0780)	(0.0816)	(0.0851)	(0.108)	(0.137)
edu= 3	−0.121	−0.122	−0.155 *	−0.00223	−0.0816
	(0.0828)	(0.0854)	(0.0904)	(0.106)	(0.134)
edu= 4	−0.0291	−0.0801	−0.192 *	0.0390	0.0911
	(0.0989)	(0.102)	(0.109)	(0.118)	(0.143)
edu= 5	−0.337 **	−0.472 ***	−0.635 ***	−0.436 **	−0.154
	(0.157)	(0.160)	(0.176)	(0.187)	(0.220)
edu= 6	−0.155	−0.0758	−0.451	-0.387	0.158
	(0.530)	(0.591)	(0.668)	(0.671)	(0.709)
marry= 1	0.379 ***	0.326 ***	0.468 ***	−0.000523	0.337 **
	(0.0813)	(0.0846)	(0.0900)	(0.122)	(0.164)
ifpension= 1	0.186 ***	0.198 ***	0.230 ***	0.130	0.204 *
	(0.0639)	(0.0666)	(0.0708)	(0.0845)	(0.108)
ifland= 1	0.623 ***	0.644 ***	0.874 ***	0.121	0.335 ***
	(0.0615)	(0.0637)	(0.0672)	(0.0812)	(0.101)
ifmachine= 1	0.620 ***	0.643 ***	0.763 ***	0.476 ***	0.228 **
	(0.0621)	(0.0644)	(0.0684)	(0.0825)	(0.103)
pexp(×10^−4^)	−0.369 **	−0.360 **	−1.04 ***	−0.443 *	0.435 *
	(1.79 × 10^−5^)	(1.83 × 10^−5^)	(2.56 × 10^−5^)	(2.60 × 10^−5^)	(2.31 × 10^−5^)
nagrincr	−0.401 ***	−0.391 ***	−0.567 ***	0.00153	−0.357 ***
	(0.0759)	(0.0792)	(0.0719)	(0.0434)	(0.121)
region= 2	−0.879 ***	−0.909 ***	−0.880 ***	−0.938 ***	−0.756 ***
	(0.0959)	(0.0992)	(0.103)	(0.138)	(0.165)
region= 3	−0.274 ***	−0.325 ***	−0.421 ***	0.0416	−0.167
	(0.0680)	(0.0710)	(0.0729)	(0.0934)	(0.115)
region= 4	−0.343 ***	−0.520 ***	−0.839 ***	0.206 **	−0.238 **
	(0.0713)	(0.0756)	(0.0798)	(0.0958)	(0.120)
cons	0.949	0.370	−2.985 **	−4.155 **	5.671 ***
	(1.123)	(1.173)	(1.231)	(1.872)	(2.071)
LR Chi2	1788.61 ***	1655.47 ***	3495.12 ***		

Note:(1) Standard error is in parentheses; (2) *, ** and *** denote 10%, 5% and 1% significance levels; (3) In both the logit and multinomial logit regression, the baseline group consists of the people not working.

**Table 3:** Labour time decision of rural elderly people.

Variables	Heckman	BFG		
	(1) Overall	(2) Agricultural Employment	(3) Non-Agricultural Employment	(4) Non-Agricultural Self-Employment
psah	−149.2 ***	32.27	114.77	−96.12
	(31.28)	(54.56)	(155.65)	(263.05)
age	256.4	651.61	−1329.44	−3533.97 *
	(265.1)	(453.52)	(1594.70)	(1937.36)
agesq	−40.98 *	−57.76	147.69	288.46 **
	(22.80)	(35.51)	(138.44)	(142.96)
gender= 2	−268.8 ***	−148.63	718.41 **	494.09
	(42.56)	(98.37)	(302.41)	(466.48)
edu= 2	0.0149	58.54	−151.47 *	272.70
	(45.21)	(45.77)	(91.43)	(165.45)
edu= 3	−45.48	14.85	−91.8899	101.55
	(45.80)	(48.36)	(80.67)	(151.00)
edu= 4	34.93	41.96	−65.94	197.38
	(48.81)	(63.59)	(98.84)	(201.89)
edu= 5	193.8 **	165.12	177.49	528.42 *
	(82.22)	(103.80)	(156.52)	(272.11)
edu= 6	115.2	−374.30	340.01	348.70
	(258.4)	(362.26)	(470.69)	(717.07)
ifpension= 1	−72.55 *	−72.2121	−77.20	19.54
	(38.64)	(46.90)	(86.06)	(169.35)
Landarea	0.197	0.9342 ***	−0.29	0.27
	(0.229)	(0.32)	(0.30)	(1.02)
ifmachine= 1	31.66	147.90 ***	−205.84	−429.78 **
	(35.60)	(49.36)	(136.89)	(195.54)
Pexp	0.00111	−0.003	0.001	0.007
	(0.001)	(0.002)	(0.004)	(0.007)
region= 2	−199.7 ***	−145.34 **	−61.05	261.43
	(65.10)	(72.09)	(186.15)	(258.21)
region= 3	−161.3 ***	−219.33 ***	−250.99 ***	−117.80
	(38.83)	(41.98)	(94.08)	(151.04)
region= 4	47.65	−138.61 **	−318.284 **	−314.70
	(44.98)	(62.10)	(153.95)	(204.85)
Cons	1784.4 **	−270.86	5498.16	7664.64 **
	(767.3)	(1617.3)	(5358.2)	(3657.3)
Wald Chi2/F test	20.45 ***	6.74 ***	4.61 ***	1.96 **

Note:(1) Standard error is in parentheses; (2) *, ** and *** denote 10%, 5% and 1% significance levels.

**Table 4:** The gender and age differences of health impaction of LFP.

Groups	(1) Overall		(2) Agricultural Employment		(3) Non-Agricultural Employment		(4) Non-Agricultural Self-Employment	
	Coefficient	ME	Coefficient	ME	Coefficient	ME	Coefficient	ME
male	−0.812 ***	−0.0874	−0.669 ***	0.0160	−1.140 ***	−0.0926	−0.918 ***	−0.0118
female	−0.487 ***	−0.0869	−0.401 ***	−0.0192	−0.728 ***	−0.0455	−0.770 ***	−0.0236
45–50	−0.544 ***	−0.0521	−0.418 ***	0.0293	−0.777 ***	−0.0718	−0.629 ***	−0.0126
51–55	−0.598 ***	−0.0668	−0.446 ***	0.0262	−0.751 ***	−0.0544	−0.878 ***	−0.0399
56–60	−0.713 ***	−0.0943	−0.531 ***	0.0337	−1.157 ***	−0.1079	−1.041 ***	−0.0225
61–65	−0.567 ***	−0.0871	−0.453 ***	0.0016	−0.997 ***	−0.0875	−0.559 ***	−0.0035
66–70	−0.710 ***	−0.1267	−0.621 ***	−0.0469	−1.019 ***	−0.0544	−1.053 ***	−0.0270
>70	−0.522 ***	−0.1109	−0.512 ***	−0.0891	−0.692 ***	−0.0170	−0.414 **	−0.0045

Note: (1) ME is the marginal effect on mean; (2) *, ** and *** denote 10%, 5% and 1% significance levels.

**Table 5:** The gender and age differences of health impaction of labor time.

Labor Time	(1) Overall	(2) Agricultural Employment	(3) Non-Agricultural Employment	(4) Non-Agricultural Self-Employment
Male	−163.4 ***	−48.13	36.68	44.06
Female	−106.9 **	56.94*	−24.98	−321.5
45–50	−192.8 **	−23.77	149.2	61.97
51–55	−205.5 ***	30.1	13.28	107.6
56–60	−254.9 ***	36.85	239.1	542.7
61–65	−200.0 ***	−32.28	−124.7	−180.5
66–70	−84.75	90.24	−471.8 *	−2287
>70	19.6	33.68	209.1	−87.65

Note: *, ** and *** denote 10%, 5% and 1% significance levels.

## Appendix A

**Table A1:** The description of objective health.

Variable Name	Definitions	Value Description
Disab (1–5)	has this type of functional disability: (1) physical disability, (2) brain damage (or mental retardation), (3) blindness (or semi-blindness), (4) paralysis (or semi-squatting), (5) dumbness (or severe stuttering)	0 no, 1 yes
Chronic (1–14)	has chronic disease: (1) high blood pressure, (2) dyslipidaemia, (3) diabetes or elevated blood sugar, (4) malignant tumours such as cancer, (5) chronic lung disease, (6) liver disease, (7) heart disease, (8) stroke, (9) kidney disease, (10) stomach or digestive diseases, (11) emotional and mental problems, (12) memory-related diseases, (13) arthritis or rheumatism, (14) asthma	0 no, 1 yes
bodypains	has any pain or discomfort in the body	0 no, 1 yes
otherdisease	has other diseases (in addition to disability and chronic diseases)	0 no, 1 yes
adls (1–12)	the difficulty level of completing this activity: (1) 1 km running or jogging, (2) standing sedentarily, (3) climbing stairs, (4) bending, knees or squat, (5) stretching along the arm, (6) lifting 5 kg weights, (7) picking up a small coin from a table, (8) housework, (9) cooking (10) going to shops to buy groceries, (11) managing money, (12) taking medicine	1 has no difficulty, 2 has little difficulty and can complete, 3 has more difficulty and needing help, 4 cannot complete
cesd	the mean value of Center for Epidemiologic Studies Depression Scale (CES-D)	has 3 degrees (1–3) in each scale: the greater the value, the higher degree of psychological depression
